# On the Structure of Oxygen Deficient Amorphous Oxide Films

**DOI:** 10.1002/advs.202306243

**Published:** 2023-12-26

**Authors:** Jack Strand, Alexander L. Shluger

**Affiliations:** ^1^ Department of Physics and Astronomy University College London Gower Street London WC1E 6BT UK; ^2^ Nanolayers Research Computing Ltd. London UK; ^3^ WPI‐Advanced Institute for Materials Research (WPI‐AIMR) Tohoku University 2–1‐1 Katahira, Aoba‐ku Sendai 980‐8577 Japan

**Keywords:** amorphous oxide, computer modeling, defects

## Abstract

Understanding defects in amorphous oxide films and heterostructures is vital to improving performance of microelectronic devices, thin‐film transistors, and electrocatalysis. However, to what extent the structure and properties of point defects in amorphous solids are similar to those in the crystalline phase are still debated. The validity of this analogy and the experimental and theoretical evidence of the effects of oxygen deficiency in amorphous oxide films are critically discussed. The authors start with the meaning and significance of defect models, such as “oxygen vacancy” in crystalline oxides, and then introduce experimental and computational methods used to study intrinsic defects in amorphous oxides and discuss their limitations and challenges. To test the validity of existing defect models, ab initio molecular dynamics is used with a non‐local density functional to model the structure and electronic properties of oxygen‐deficient amorphous alumina. Unlike some previous studies, the formation of deep defect states in the bandgap caused by the oxygen deficiency is found. Apart from atomistic structures analogous to crystal vacancies, the formation of more stable defect states characterized by the bond formation between under‐coordinated Al ions is shown. The limitations of such defect models and how they may be overcome in simulations are discussed.

## Graphical abstract

Defects in amorphous oxide films and heterostructures affect the performance ofmicroelectronic devices, thin‐film transistors, and electrocatalysis. In this perspective, the experimental and theoretical evidence of the effects of oxygen deficiency in amorphous oxide films and the validity of the analogy between the structure and properties of point defects in amorphous and crystalline oxides are critically discussed.

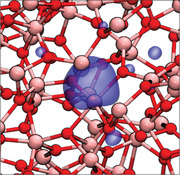

## 1 Introduction

**Figure 1:**
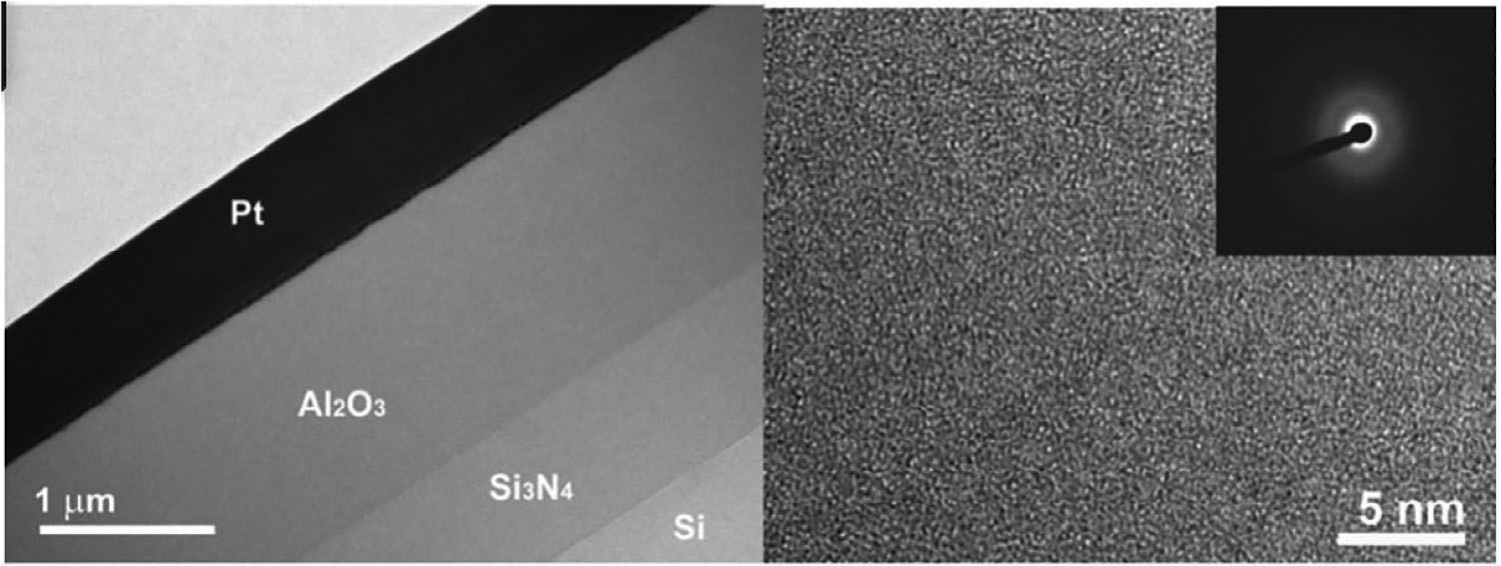
Cross section TEM image (left) of the Pt/a‐Al_2_O_3_/Si_3_N_4_/Si heterostructure and high resolution TEM image of the a‐Al_2_O_3_ film (right) with a diffraction pattern (inset). Layered heterostructures such as this a common to many nanoscale technologies. Reproduced with permission.^[^ ^9^ ^]^ Copyright 2009, The American Physical Society.

Non‐crystalline solids are usually defined as those lacking the long‐range order characteristic of a crystal. Glass is one prominent and relatively well‐defined example of a non‐crystalline solid. It can be obtained by cooling a liquid fast enough so that a more standard first‐order phase transition toward the crystalline phase is avoided.^[^ ^1^, ^2^, ^3^ ^]^ Amorphous oxide films and heterostructures are another fast‐emerging example of non‐crystalline solids.^[^ ^4^ ^]^ Many binary and more complex oxides do not undergo a glass transition but form disordered, non‐crystalline semiconducting^[^ ^5^, ^6^, ^7^ ^]^ or insulating films when grown or deposited on substrates.^[^ ^8^ ^]^ **Figure** **1** shows an example of such a heterostructure where the amorphous Al_2_O_3_ and Si_3_N_4_ films are sandwiched between the Si and Pt electrodes.^[^ ^9^ ^]^ As evidenced by X‐ray diffraction and the lack of granular structure in the TEM image, the structure of the Al_2_O_3_ film is amorphous. The Si and Pt electrodes are used to apply electrical bias, which may be accompanied by the injection of electrons or holes into the insulating films. Such structures are common in memory applications. Here, we do not discuss the important role of substrates and interfaces (see, for example refs. [10, 11]) and focus on the bulk properties of amorphous oxides.

**Figure 2:**
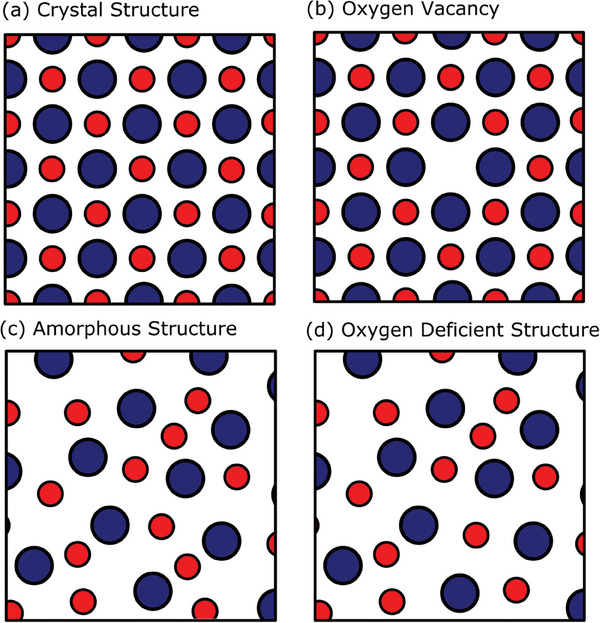
Schematic crystalline and amorphous perfect and defective structures. a) Plane projection of *fcc* structure of a binary ionic oxide (oxygen ions are shown in red). b) The same *fcc* structure with one oxygen ion missing. Since the lattice is well‐defined, missing oxygen atoms (“vacancies”) are easy to identify. c) A schematic of amorphous structure of the same solid. d) The same structure with one oxygen ion missing. The density is reduced with respect to crystal and ions have on average lower coordinations. Due to disorder, it is more difficult to identify the position of the missing oxygen ion.

Amorphous oxide films are metastable and tend to crystallize upon annealing at high temperatures. However, they are cheaper to grow and have advantageous properties in manufacturing and applications in flexible electronics and many other technologies as dielectrics (e.g., SiO_2_, HfO_2_, Al_2_O_3_) and semiconductors (indium gallium zinc oxide (IGZO), Ga_2_O_3_, Ta_2_O_5_, and others).^[^ ^7^, ^8^, ^11^ ^]^ Some of these oxides, such as Al_2_O_3_ and ZrO_2_, are also classified as “intermediate glass formers” because they can be both a glass former and a glass modifier in binary oxide glasses but cannot solely form glass.^[^ ^12^, ^13^ ^]^ Their performance in electronic devices is affected by electrical and structural degradation, attributed to their metastable nature, as well as to pre‐existing and generated point defects.^[^ ^6^, ^14^ ^]^ Among these defects, oxygen vacancies are routinely alleged to cause many effects degrading the performance of electronic devices (e.g., field effect transistors and memory cells)^[^ ^14^, ^15^ ^]^ often by analogy with crystals. However, the nature and structure of even these basic defects in amorphous oxides remain poorly understood due to the absence of long‐range order and variations in local order. Unlike crystal defects, which are defined naturally as a violation of regular crystallographic order, such as a missing or substitution atom in a lattice site, defects in disordered systems are more difficult to define because of the lack of such an order. In addition, defects can cause further significant rearrangements and distortions of the local structure and adopt several non‐equivalent configurations. The familiar terminology, that is used to describe defects in scientific discussions, such as “oxygen vacancy with an unpaired electron,” “interstitial oxygen atom forming a dumbell configuration with another oxygen ion in a lattice site,” or “impurity ion substituting cation in a lattice site,” refers to atomic sites in crystal structures. However, the positions of missing or additional atoms in amorphous structures are defined only with respect to their close neighbors rather than the periodic lattice. This is further illustrated in **Figure** **2** where we compare the perfect and defective structures of crystalline and amorphous solids. Although defect models have been routinely transferred from crystals to amorphous solids, the validity of this correspondence has not been justified. Structural disorder has different consequences in metals, covalent semiconductors, and ionic insulators. In this perspective, we focus on recent developments in our understanding of the structure and properties of defects in oxygen‐deficient amorphous oxide films and whether we can call them oxygen vacancies. Addressing this issue requires going beyond models assuming similarity to well‐defined crystallographic vacancies.

Below, we provide a brief overview of previous experiments and computational works and discuss challenges for studying the properties of defects in amorphous materials. In the next section, we discuss the meaning and significance of defect models, such as “oxygen vacancy,” and revisit how experimental and theoretical methods were used to establish oxygen vacancy models in some crystalline oxides. In Section 3, we discuss the results of experimental and theoretical studies of oxygen‐deficient amorphous oxide films. To better understand the sources of discrepancies between different calculations, an overview of computational issues in modeling defects in amorphous oxides is given in Section 4. We then attempt to elucidate some of these issues via re‐examining the nature of oxygen vacancies in amorphous alumina. Our results demonstrate that the results of theoretical simulations are very sensitive to calculation methods and the initial amorphous structures. Structural disorder may have different consequences in different oxides, for example, IGZO and the wider‐gap alumina. Specifically, in amorphous alumina we find that defects induced by oxygen deficiency are stable and form deep electron states, however, their atomic structure depends on the way they have been created, sample history, and charge state. Apart from point defects structurally similar to oxygen vacancies in the crystal phase, we show the formation of more stable defect states characterized by the bond formation between under‐coordinated Al ions. These defects are stable with respect to the structure relaxation caused by changes of the charge state, supporting some of the previous models of electron transfer in amorphous alumina films.^[^ ^16^ ^]^ We conclude that “point defect” remains a meaningful concept in amorphous oxides, but some defect configurations may have different meanings to crystallographic defects. Structural disorder of amorphous materials creates complex energy landscapes^[^ ^17^ ^]^ where atomistic structures of defects, such as vacancies, can evolve as a result of thermal annealing, bias application, carrier transport, irradiation, and other factors.

## 2 Establishing Defect Models

Defect models used in scientific literature and discussions differ by the level of details. Electrical and chemical properties of reduced oxides are often attributed to “oxygen vacancies.” However, whether oxygen vacancies or other defects are responsible for the material's properties and more detailed, *atomistic* structural models of point defects are very difficult to establish even in crystals.^[^ ^18^, ^19^, ^20^, ^21^, ^22^ ^]^ Such models in solids usually stem from the theoretical analysis and synthesis of many experimental observations, such as optical absorption and photo‐luminescence,^[^ ^19^ ^]^ electron paramagnetic resonance (EPR),^[^ ^23^, ^24^ ^]^ and X‐ray absorption spectroscopy,^[^ ^25^ ^]^ combined with theoretical modeling.^[^ ^21^ ^]^

To illustrate this point, let us briefly recall how defect models were developed in reduced ionic crystal MgO. High‐purity samples of MgO crystals grown by the arc‐fusion method at Oak Ridge National Laboratory have a known impurity content.^[^ ^26^ ^]^ When subjected to thermochemical reduction in Mg vapor, also called additive coloration, which involved heating to 2200 K for 2 h in 6 atm of Mg vapor, or to neutron irradiation, they exhibit a wide optical absorption band peaking at about 5.0 eV,^[^ ^27^ ^]^ but no EPR signal. Further irradiation with X rays induces an EPR signal showing a clearly resolved nuclear hyperfine interaction with one or two ^25^Mg nuclei.^[^ ^28^ ^]^ The analysis of these results is interpreted as the formation of oxygen vacancies containing one unpaired electron. The degree of localization of this electron can be deduced from the magnitude of the hyperfine interaction of the electron with the nearest‐neighbor Mg nuclei using a spin‐hamiltonian analysis.^[^ ^26^ ^]^ Further, the analysis of the electron‐nuclear double‐resonance (ENDOR) spectra^[^ ^26^ ^]^ has provided information regarding the outward displacements of the nearest neighbor Mg ions. By analogy with halogen vacancies in alkali halides,^[^ ^19^ ^]^ these defects have been termed *F* ^+^ (*F* for German *Farbe*) centers^[^ ^28^ ^]^ and have been assigned a *qualitative* model of an electron localized at a vacant site of an O atom in the MgO lattice. The neutral counterpart of the *F* ^+^ center is believed to contain two electrons in an O vacancy and is called a *F* center. Both centers exhibit photoluminescence, the mechanism of which remains controversial due to the reactions of hydrogen with *F* centers in the samples (see, e.g., refs. [29, 30]). Thus, analysis of the EPR and ENDOR spectra of reduced MgO crystals gave rise to a simple defect model in which an oxygen atom is removed from the crystal site as a result of thermochemical reduction or irradiation. Photoconversion gives rise to two charge states of the defect where one or two electrons are associated with the vacancy.^[^ ^29^ ^]^

Since the 1970s, *F* centers in the bulk and at the surface of MgO have become a testbed for theoretical calculations using semi‐empirical,^[^ ^31^ ^]^ Hartree‐Fock,^[^ ^32^, ^33^ ^]^ DFT,^[^ ^34^, ^35^ ^]^ quantum Monte‐Carlo,^[^ ^36^ ^]^ and multireference density matrix embedding theory,^[^ ^37^ ^]^ to mention a few initial studies. Such calculations use a set of coordinates for atomic positions. In the case of a crystal, these coordinates are determined by the crystal structure and when periodic boundary conditions are applied, only the coordinates of the chosen unit cell are necessary to form the model of the bulk, defect‐free crystal. For an *atomistic* defect model, the objective is to find the set of atomic positions that accurately reflect the atomic structure and properties of a defective system in its given charge state and mutliplicity. In the case of an oxygen vacancy, for example, the objective may be to find a set of atomic positions in a crystal with one missing oxygen atom, corresponding to the lowest potential energy. Therefore, all calculations were performed by removing an O atom from a regular lattice site in a cluster of atoms or a periodic cell and calculating the electronic structure and geometry of the remaining system at 0 K. These calculations provide a wealth of information regarding the electronic structure of a defective crystal, the defect‐induced distortion of the lattice, and further allow testing of the defect model. These calculations also predict the existence and stability of a doubly charged O vacancy in MgO and other oxides (see e.g. refs. [35, 36, 38]), an *F* ^2 +^ center. This simplest diamagnetic defect does not have occupied electronic states in the band gap and therefore is difficult to detect experimentally. Its structure is shown schematically in Figure 2b. Such vacancies can diffuse into the bulk of an oxide from surfaces, edge dislocations, or grain boundaries, and manifest themselves as charge compensators of impurity ions, in photoconversion reactions, ion (self) diffusion, and as electron traps.

Similar experimental EPR studies and theoretical calculations carried out in cubic CaO and SrO^[^ ^26^ ^]^ confirmed the *F* ^+^ center model. The formation of *F* ^+^ centers has been shown using EPR also in neutron and γ‐irradiated high‐purity α‐corundum Al_2_O_3_ crystals^[^ ^39^, ^40^ ^]^ as well as in other oxide crystals, such as LiAlO_2_, LiNbO_3_, and many more.^[^ ^38^, ^41^ ^]^ Thus, since 1950s, the existence of oxygen vacancies in oxide crystals in three charge states and the *F* center model have an established concepts. This model has been transferred to monoclinic HfO_2_,^[^ ^42^, ^43^ ^]^ where it has also been shown to have two negative charge states.^[^ ^44^ ^]^ However the *F* center type oxygen vacancy model, where the maximum of electron density is in the middle of the vacancy, is not universal, and oxygen vacancies have different structures in SiO_2_^[^ ^45^ ^]^ in TiO_2_, CeO_2_, V_2_O_5_, and other oxides.^[^ ^46^ ^]^ Kröger and Vink suggested^[^ ^47^ ^]^ a more general set of conventions to describe the relative electric charges and lattice positions of point defect species in crystals. Using this convention, these centers are $V_{O}^{\times }$ and $V_{O}^{\cdot }$ for *F* center and *F* ^+^ center, respectively, again using the concept of lattice site. We note that the electronic charge of the species is calculated relative to the site that it occupies. The charge of the species is calculated by the charge on the current site minus the charge on the original site.

Below, we discuss whether these defect models can be transferred to *amorphous* oxides, such as Al_2_O_3_ and HfO_2_. How does nonstoichiometry manifest itself in amorphous networks where the notion of an atomic “site” is defined only in theoretical simulations that operate with atomic coordinates? This point is illustrated in Figure 2, where we schematically show a 2D projection of an fcc crystalline ionic lattice with one missing ion and the same system with disordered positions of the ions. One can see that the disorder makes structural identification of the vacant site more difficult. In covalent networks, defects are often defined in terms of different coordination numbers, such as dangling bonds. This view has recently been challenged in extensive calculations of amorphous Si.^[^ ^48^ ^]^ Defect models can also be identified by analyzing structures responsible for localized states with energies in the band gap.^[^ ^49^ ^]^ However, this identification is not unique either, as most defect structures are metastable and depend on the method of creation in both experimental and theoretical studies. In the next section, we briefly review defect models in several amorphous oxides to demonstrate this point more clearly.

## 3 Oxygen Deficiency in Amorphous Oxides

Different types of oxygen‐based defects can form during the growth of oxide films at low oxygen pressure, due to oxygen scavenging by metal films during high‐*T* anneal, as a result of irradiation by neutrons or ionizing radiation, and due to application of electrical bias and injection of electrons or holes from electrodes in the device operation process.^[^ ^14^, ^19^, ^50^, ^51^ ^]^ However, the amount of experimental data characterizing point defects in amorphous oxide films is limited, and the available data are much less detailed than in the case of bulk crystals.^[^ ^6^, ^15^, ^52^ ^]^ Therefore, the properties of these defects and their atomic structures are very difficult to establish experimentally using techniques effective in bulk crystals because their sensitivities (e.g., optical absorption, EPR, EXAFS, etc.) are not sufficient to probe defect properties in nanometer‐thin films used in many devices. In fact, most of the information on defects in thin amorphous oxide films stems from analysis of electrical measurements and theoretical simulations.^[^ ^53^ ^]^ Electron and hole trapping by defects and transport through defects in oxide films are the origin of many phenomena plaguing the performance of devices, such as film charging, random telegraph noise, bias temperature instability, and dielectric breakdown.^[^ ^14^, ^15^ ^]^ In these processes, electrons (holes) are transferred to/from electrodes changing the defect charge state. Hence, the ability of defects in amorphous films to reversibly trap/detrap electrons is a fundamental property at the center of device performance and reliability. Electrical measurements of current through oxide films or changes in capacitance are extremely sensitive and can detect even single‐electron processes.^[^ ^15^ ^]^ However, unambiguously establishing the nature and atomistic structures of the electron traps responsible for these processes can be very complex.^[^ ^15^, ^53^ ^]^ The difficulty of experimental characterization of amorphous oxide films and the increased power of computation lead to a heavy reliance on atomistic simulations to understand defects in amorphous oxides and to predict their behavior. Many of such simulations involve significant approximations, and linking their predictions with experimental observations is often a challenge.^[^ ^21^, ^53^ ^]^

**Figure 3:**
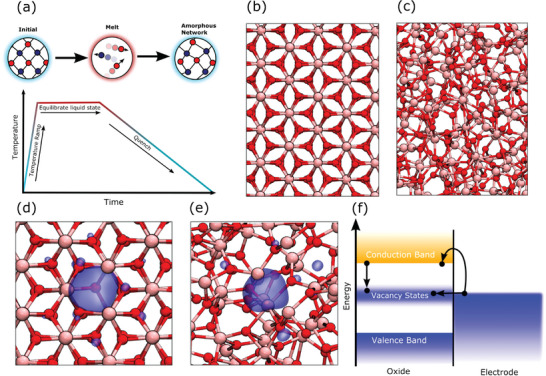
a) Schematic of the melt‐and‐quench process use to generate pseudo‐amorphous models. b) The α‐phase of Al_2_O_3_ (corundum). c) A sample model of a‐Al_2_O_3_. d) A model of an oxygen vacancy in α‐Al_2_O_3_. e) A model of an oxygen vacancy in a‐Al_2_O_3_. The blue surface is an iso‐surface of the |ψ|^2^ of the in‐gap one‐electron state of the vacancy. f) Schematic of the band allignment of an oxide containing oxygen vacancies with a metal electrode. Defects, such as vacancies, produce in‐gap states. Electrons from the conduction band or from a nearby electron source (e.g., electrode) can transfer into the vacancy states via capture (from the conduction band) or tunnelling (from an electrode). Peach ions indicate Al, red ions indicate O.

As mentioned earlier, modeling defects in amorphous solids has been often carried out by analogy with their crystalline counterparts. In particular, in many previous theoretical studies of amorphous oxides, defect models have been created using a “pick and relax” (P&R) method (see, for example, refs. [6, 42, 54, 55, 56, 57]) following the language and principles borrowed from crystal physics. For example, oxygen vacancies in wide‐gap amorphous insulators (a‐SiO_2_,^[^ ^54^ ^]^ a‐HfO_2_,^[^ ^42^ ^]^ a‐Al_2_O_3_^[^ ^58^ ^]^) have been created by removing atoms from different sites in amorphous networks, accompanied by their geometry optimization at 0 K. This workflow is further illustrated in **Figure** **3**. Models of amorphous structures were created using the so‐called melt‐and‐quench (M&Q) method^[^ ^59^ ^]^ schematically illustrated in Figure 3a. This method mimics the process used in the production of simple glasses, as discussed in more detail below. Figure 3b,c compares the structures of crystalline corundum and amorphous alumina, Al_2_O_3_. The amorphus structure has lower density, no long‐range order, and is characterized by distributions of coordination numbers of O and Al ions. In simulations, such distributions are obtained by studying a statistical set of unique amorphous models (see the discussion below). To model oxygen‐deficient structures, an oxygen atom is usually removed from random sites in each structure, and the geometry is optimized again. The defect structures obtained in this way in Al_2_O_3_ are broadly similar in both phases, as can be seen in Figure 3d,e. They exhibit a strong localization of two electrons in the potential well created at the vacant oxygen site. However, the structure and properties of O vacancies in the amorphous phase depend on the local environment.^[^ ^58^ ^]^ Unlike in crystals, the one‐electron and charge transition levels in the amorphous phase exhibit wide energy distributions, which are probed in P&R simulations by removing oxygen atoms at different network positions and in several different amorphous structure models (see, e.g., discussion in ref. [60]). The distribution of defect states in the band gap is schematically illustrated in Figure 3f, where we also show that these defect states should be considered in the context of interfaces with metallic or semiconducting substrates. In devices, electrons or holes can be injected from electrodes into these defect states, causing changes in their occupation.

In many studies, the existence of oxygen vacancies in amorphous films and silica glass and their ability to reversibly trap and release electrons is taken for granted.^[^ ^53^, ^57^ ^]^ This intuition stems from observations that the electrical,^[^ ^61^, ^62^, ^63^, ^64^ ^]^ optical,^[^ ^65^, ^66^ ^]^ and mechanical^[^ ^67^ ^]^ performance of poly‐crystalline and amorphous oxide films of the same composition is qualitatively similar. However, to what extent the analogy between the structures and electronic properties of defects in crystalline and amorphous oxides can be stretched remains unclear. Several recent papers^[^ ^5^, ^68^, ^69^, ^70^ ^]^ question this notion and ask profound questions: How to define a defect in a disordered structure and whether such defects can exist at all in the conventional sense? Let us consider several examples.

In fully coordinated a‐SiO_2_, a neutral O‐vacancy is usually associated with the formation of a Si–Si bond at the place of the missing O atom and is characterized by the optical absorption band at 7.6 eV both in α‐quartz and in fused silica glass.^[^ ^71^ ^]^ The theoretical calculations^[^ ^72^, ^73^ ^]^ predict that the vacancy formation causes a very long‐range distortion of the surrounding silica network; however, the details of network distortion caused by O vacancies created by the P&R method differ from those obtained using the high‐*T* anneal.^[^ ^73^ ^]^ This is caused by the relaxation of the amorphous network during anneal and suggests that static and molecular dynamics simulations of defect structures in amorphous structures can lead to different conclusions. The trapping of holes by neutral vacancies leads to the formation of a family of paramagnetic defects designated *E*′ centers, based on their EPR signals.^[^ ^74^, ^75^ ^]^ The models of these defects in a‐SiO_2_ are more diverse than in the crystalline phase α‐quartz.^[^ ^54^, ^55^, ^71^, ^76^ ^]^ The theoretical calculations have predicted the existence of five charge states of the O vacancy in a‐SiO_2_ (with defect charges ranging +2…0…−2),^[^ ^77^, ^78^, ^79^ ^]^ as well as more than eight different geometric configurations for the +1 charge state vacancy in a‐SiO_2_ (see, e.g., refs. [76, 78]). When transforming between these charge states and configurations, oxygen deficiency centers in SiO_2_ undergo lattice relaxation with atomic displacements often exceeding 0.5 Å,^[^ ^78^ ^]^ in excess of typical displacements in other oxides (0.1—0.3 Å ^[^ ^46^ ^]^). The abundance of predicted configurations of positively charged vacancies in a‐SiO_2_ stems from the flexibility of the SiO_2_ network of corner sharing tetrahedra and different short and medium range order environments surrounding defects due to disorder. Several of these configurations have not been detected experimentally using EPR, most likely because they are metastable or require overcoming high barriers for their formation (see discussion in ref. [80]). However, there is no doubt about the existence of both neutral and positively charged oxygen vacancies in bulk crystalline and amorphous SiO_2_.

In more ionic oxides, such as HfO_2_ and Al_2_O_3_, the situation is different. The amorphous structures of these oxides are characterized by the distribution of how many nearest‐neighbor ions surround a particular metal or oxygen ion. For example, unlike the crystal phase of corrundum Al_2_O_3_, where Al is coordinated by six O ions, in the amorphous phase, the Al ions are 4, 5, or 6‐coordinated.^[^ ^81^ ^]^ In such systems, the definition and characterization of vacancies and interstitial atoms are more complicated.^[^ ^68^, ^82^ ^]^ In amorphous Al_2_O_3_, the electron energy loss spectra of atomic layer‐deposited films have demonstrated the existence of a 6.4 eV peak,^[^ ^83^ ^]^ which has been attributed using DFT simulations to a neutral oxygen vacancy. Electron trapping in amorphous alumina films^[^ ^84^ ^]^ and electron conduction through amorphous alumina films^[^ ^85^ ^]^ are also attributed to the presence of oxygen vacancies in these films. Oxygen scavenging by metal films can lead to oxygen deficiency in oxide. This has been demonstrated at the interface of the TiN film with crystalline γ‐Al_2_O_3_.^[^ ^86^ ^]^ However, the data on oxygen scavenging by Ti in Ti/a‐Al_2_O_3_/InGaAs gate stacks^[^ ^87^ ^]^ suggest that there is no driving force for the dissociation of a‐Al_2_O_3_ by the thin Ti layer and hence oxygen scavenging from a‐Al_2_O_3_. On a theoretical front, the static DFT calculations using the P&R method predict the similar structures and properties for O vacancies in crystalline and a‐Al_2_O_3_.^[^ ^58^ ^]^ However, similar calculations in ref. [88] predict the existence of two distinct types of atomic and electronic structures for neutral O vacancies with an energy barrier between them in a‐Al_2_O_3_. The DFT molecular dynamics simulations,^[^ ^68^ ^]^ on the other hand, predict that oxygen vacancies do not occur in amorphous Al_2_O_3_, due to structural rearrangements, which “assimilate” the defect structure and cause a delocalization of the associated defect levels. Therefore, both experimental and theoretical evidence for the existence and structure of oxygen vacancies in a‐Al_2_O_3_ is still inconclusive.

Thorough theoretical investigations carried out in amorphous InGaZnO_4_ (IGZO)^[^ ^69^, ^70^, ^89^ ^]^ and In_2_O_3 − *x*_^[^ ^90^ ^]^ also came to conflicting conclusions regarding the character and stability of oxygen vacancies. In particular, the results of calculations by refs. [69, 70] demonstrate that the +2 charged vacancy “blends” into the amorphous structure. This means that it cannot be structurally identified in the amorphous structure and geometrically distinguished from a low‐coordinated cation, as schematically illustrated in Figure 2c,d. However, after two electrons are added to the system, the neutral vacancy state with two electrons localized inside the vacancy is restored in ref. [69] but the vacancy remains “ionized” and the added electrons are delocalized in the conduction band in ref. [70]. A similar contradiction in the behavior of O vacancy in amorphous HfO_2_ can be noted in refs. [42, 91] and [82].

Clearly, how oxygen deficiency manifests itself and whether an oxygen vacancy can serve as an electron trap in a‐Al_2_O_3_ and other amorphous oxides remain unclear. The experimental evidence is circumstantial, and the theoretical results are controversial. However, only theoretical modeling can provide atomistic structural models of oxygen deficient amorphous structures. To better understand the theoretical predictions, one needs to look deeper into how the models and methods used in these calculations affect the results. Therefore, below we briefly discuss some of the limitations of computational methods used to simulate defective amorphous oxides and then re‐visit the modeling of one such system—oxygen vacancies in amorphous Al_2_O_3_. Amorphous Al_2_O_3_ is representative of other intermediate glass formers, such as ZrO_2_ and HfO_2_, and has wide applications, and this insight may prove useful for understanding other systems too.

## 4 Simulation Techniques

In the conventional P&R method, an oxygen atom is removed from a chosen site in a pseudo‐amorphous cell. Thus, the defect is defined with respect to the original amorphous structure. But how reliable are theoretical models of amorphous structures? The density, thickness, and morphology of oxide films are strongly dependent on the substrate material and the method of deposition. This makes simulation of these structures challenging (see refs. [57, 59] for discussion). Here, we briefly outline the main approximations important for our discussion of intrinsic defects in amorphous oxides.

First, we should differentiate between the bulk and interface models. Most of the systems described in this paper consider bulk amorphous oxides, although they pertain to thin amorphous films. Modeling interfaces is made complex by issues such as lattice mismatch and the need for a “connection scheme” joining atoms at the interface, and interface calculations are usually computationally intensive. Interface models are therefore prohibitively complex for our inquiry. The effects of interfaces with Si and other substrates have been simulated in a number of papers (see, e.g., refs. [92, 93, 94]). These are important but require a separate review. Second, all computational models of amorphous structures use 3D periodic boundary conditions to exclude surface effects. Therefore, these models are periodic and pseudo‐amorphous, with an amorphized cell periodically translated (see, Figure 3b,c). This results in a significant dependence of structural parameters, such as distributions of sites with different coordination, density, distribution of bond lengths, and other parameters, on the cell size (see, e.g., ref. [91]).

The most common method for creating amorphous atomic structures is based on melting crystalline samples using 3D periodic boundary conditions and the subsequent cooling of the melt at different rates to low temperatures. This so‐called melt‐and‐quench, M&Q method,^[^ ^5^, ^59^, ^95^ ^]^ is outlined in Figure 3c. This method is based on the assumption that all liquids can be frozen into an amorphous state by fast cooling. The formation of real glasses from melt has been studied extensively.^[^ ^96^ ^]^ By using this approach, we completely ignore the complexities introduced by film growth, but it has advantages too. The melt properties of some oxides, such as HfO_2_ and Al_2_O_3_, are better known than those of the amorphous phase.^[^ ^97^, ^98^ ^]^ This allows us to test computational methods. 3D periodic boundary conditions help to eliminate the nucleation of crystal phases. However, the cooling rate is usually unrealistically high due to computer limitations and is rarely lower than 0.1 K ps^−1^. Despite high cooling rates, these methods have been reasonably good at predicting densities and coordination number distributions of disordered oxides, where the experimental values have been available.^[^ ^98^, ^99^, ^100^ ^]^ When AIMD is used to create amorphous structures, the cooling rates typically used in previous studieshave been as high as 500 K ps^−1^.^[^ ^5^ ^]^ Structures of frozen melts produced in this way are metastable and the introduction of defects leads to both local distortions and global structure changes. However, AIMD has important advantages over MD using classical forcefields because of a quantum mechanical description of electrons and a more physically accurate description of interactions. For example, oxygen vacancies are known to contain electrons in their neutral charge state, affecting the charge and forces on nearby ions. In a classical force‐field description, such effects are completely missed, but these can be accounted for in AIMD.

The electronic structure of amorphous cell also depends on the cell size as well as the method of calculation. Because the atomic structure is disordered, there is no **k**‐point sampling, and all electronic structure calculations are performed at **k** = 0. However, the accuracy of the electron density depends on the cell size, as in crystal calculations. Therefore, using extended cells of similar size to those reproducing the electronic properties of crystal structures in **k** = 0 calculations is a good guide (in crystal calculations, the **k** = 0 point of the Brillouine zone is equivalent to some symmetric **k** points, determined by cell extension).

The Hamiltonian used in the calculations may qualitatively affect the conclusions. The observations of refs. [68, 70] demonstrate the importance of correctly describing the ability of electrons to localize from delocalized band states into potential wells created by disorder and distortions in an amorphous network. This problem is akin to the polaron localization problem in crystals, which has a long history (see, e.g., refs. [101, 102, 103] for recent discussions). Applications of DFT to the electron localization problems are plagued by the so‐called self‐interaction error, which stems from the fact that the residual self‐interaction in the Coulomb part of the DFT Hamiltonian and that in the exchange part do not cancel each other exactly for one electron.^[^ ^104^ ^]^ This problem becomes particularly acute when local density functionals are employed to study electron localization in molecules^[^ ^105^, ^106^ ^]^ and solids.^[^ ^107^, ^108^, ^109^ ^]^ One of the popular fixes^[^ ^108^ ^]^ concerns using the so‐called non‐local exchange‐correlation (XC) functionals where a certain portion of the exact non‐local exchange is admixed to one of the common (usually semi‐local) density functionals.^[^ ^110^ ^]^ This makes calculations much more computationally expensive but does not yet fully solve the problem, as one needs to determine the amount of Hartree–Fock exchange to be included.^[^ ^99^ ^]^ There have been studies investigating procedures by which one can justify certain choices of the Hartree–Fock exchange fraction (see e.g., refs. ^[^ ^111^, ^112^ ^]^), which rely on achieving “piecewise‐linearity,” a known property of the exact XC functional. Although these do not solve every problem, they are very successful in improving the accuracy of calculations and thereby improving the representation of localized electron states.

Finally, we note that in amorphous structures, any comparison of model parameters with experiments must involve statistical sampling of different configurations to estimate the probability of existence for each particular defect configuration and build a distribution of properties, for example, formation energies. Due to the high computational costs, more than several tens of configurations are rarely considered. Therefore, it is only feasible to talk about an approximate range in which a particular property can change rather than smooth distribution (see, e.g., discussion in refs. [53, 113]).

As noted above, oxygen related defects in amorphous films can be produced at room or lower temperatures as a result of irradiation,^[^ ^114^, ^115^ ^]^ electrical stress and/or electron injection.^[^ ^57^ ^]^ In other scenarios, such as film growth, melt solidification, high‐temperature annealing of grown films, and oxygen scavenging during annealing, electrons and ions have more possibilities to explore the structure and energy landscape to find optimal positions. To model that and to go beyond the vacancy model, one can use ab  initio molecular dynamics simulations (AIMD). This has been tried in the past^[^ ^68^, ^69^, ^70^, ^90^ ^]^ but led to controversial results. In the following, we use AIMD simulations to further explore the structure of defects in a‐Al_2_O_3_ in attempt to highlight and partially address some of the issues related to modeling oxygen deficiency discussed above. The details of these simulations are outlined in the Section below.

## 5 Modeling Oxygen Deficient Amorphous Alumina

To explore further the structure of oxygen‐deficient models of Al_2_O_3_ we performed AIMD simulations at high temperature (high‐*T*) and using a non‐local density functional. To go beyond previous studies, we used three larger a‐Al_2_O_3_ periodic cells initially containing 360 atoms with three neutral O atoms removed from each of them. Thus, each structure has six electrons left behind by the three removed O atoms, which need to find their place. In a crystal, at low temperatures they occupy the vacant oxygen sites. In a melt, no similarity to well‐defined crystallographic vacancies is assumed and these electrons may become delocalized. We are interested in their behavior during cooling and formation of amorphous phase. Therefore using classical forcefields is problematic and AIMD is required to describe these electrons. The non‐local density functional is employed to describe more accurately the electron localization. Each model undergoes a M&Q procedure and to investigate how the oxygen deficiency and these electrons can be accommodated, we analyze the degree of localization of electronic states and the evolution of the system's geometric structure.

As an alternative approach, we delete three O atoms in three pre‐existing static amorphous structures and subject them to high‐*T* anneals using AIMD, similar to the approach taken in ref. [68]. This allows us to check how the O‐vacancies created by the conventional P&R method respond to annealing and compare them with the in‐melt structures. Observing differences between models can elucidate how different approaches to generating defects in amorphous solids affect the calculated properties.

### 5.1 Properties of the Melt

**Figure 4:**
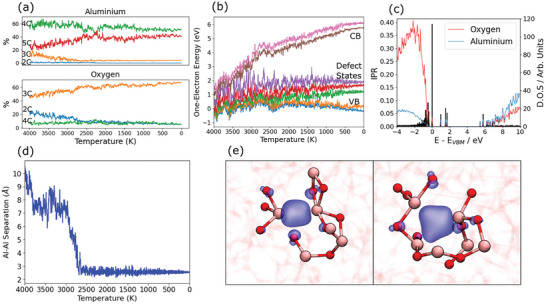
a) Coordination number frequency of Al with respect to O and O with respect to Al. In both cases, the coordination number is given as a percentage of the total number of respective ions. Both are shown for a given temperature during the quench phase. b) The one‐electron energies versus *T* during the quench phase. The energies shown correspond to the two highest energy states in the VB, the three point‐defect states in the band gap, and the two lowest energy states in the CB. To assist visibility, a 100 fs running average is used. c) IPR and PDOS of the amorphous alumina model shown in (b). One can see the one‐electron localised states in the band gap, similar to the case of oxygen vacancies in a crystal. The IPR values characterize the degree of localization of one‐electron states. d) The separation of the two Al ions, which form an Al–Al bond, during the quench phase. It can be seen that the pairing forms once the temperature is brought down to approximately 2700 K, after which the two Al ions remain bound to one another. In most cases, formation of the defect structure occurs between 3000 and 2000 K. e) Defect states formed after cooling the sub‐stoichiometric a‐Al_2_O_3_ melt. Peach spheres show Al ions, red spheres show O ions. Blue surface indicates an iso‐surface of |ψ|^2^ of an in‐gap defect state. The left image shows a 2‐coordinated defect state and the right image shows a 3‐coordinated defect state.

We start from describing the properties of the alumina melt at a range of temperatures and comparing those with the available experimental data.^[^ ^13^ ^]^ The coordination number (CN) distributions for Al and O for the range of temperatures are plotted in **Figure** **4**a In the corundum (α) phase of Al_2_O_3_, all Al ions are 6‐coordinated (6C) by oxygen ions. Other phases have mixtures of 4C and 6C Al ions. In Figure 4a, we show that a significant percentage of 5C Al ions emerge in the melt and in the amorphous solid. It can also be seen that, in the high‐*T* melt, there is a percentage of 3C Al ions, which decreases as the melt is quenched, and then a small percentage remains upon solidification. Other studies of both alumina melt at 2700 K^[^ ^98^, ^100^ ^]^ and glass^[^ ^13^ ^]^ did not report the presence of 3C Al ions, and we believe that the emergence of these ions in our samples is due to sub‐stoichiometry. Figure 4a shows that the majority of O atoms are 3C and that a significant amount of 2C oxygen ions that exist in the high‐temperature melt is reduced during the quench phase. Upon solidification, the concentration of 2C oxygen ions is approximately 10%, in agreement with other studies.^[^ ^13^, ^98^ ^]^

We note that Figure 4a exhibits a clear change in behavior at ≈2300 K. At higher temperatures, there is a strong dependence of the CN distribution on temperature, but at lower temperatures, the CN distribution remains roughly constant. This suggests that at temperatures below 2300 K there is no longer any rearrangement of topology, implying a solid structure. Our results for the CN distribution are in good agreement with experimental observations of ref. [98], where models fitted to nuclear magnetic resonance data measured at 2700 K give CNs of 63.2% for 4C Al ions and 32.2% for 5C Al ions. At low temperatures, they also agree with 55% ± 3% for 4C Al and 42% ± 3% for 5C Al reported for alumina films.^[^ ^116^ ^]^ However, we note that the reported coordination numbers vary by several percent depending on the method of film deposition and the density of the samples.^[^ ^117^, ^118^ ^]^

The electronic structure of the system undergoes striking changes during the quench phase of the simulation. Figure 4b shows a running average of the electronic structure during the quench phase. In the high‐temperature melt, there is no clear band gap. However, as the system cools and solidifies, a band gap opens and reaches 6.2–7.0 eV, characteristic of solid amorphous alumina.^[^ ^119^, ^120^ ^]^ At temperatures just below 2300 K, the band gap is still low compared to the room *T* amorphous alumina (4 eV compared to 6 eV). Most of the band gap opening during quenching arises from the change in energy of the conduction band edge, whereas the energies of the point defect states and the valence band edge remain largely constant with temperature. As the temperature decreases to 0 K, the opening of the band gap stems from the increasing density of the material, resulting in shorter inter‐atomic distances. This was checked by static calculations of the fully quenched oxide with interatomic distances scaled so as to increase the volume to a value corresponding to a particular temperature in the quench phase.

### 5.2 In‐Melt Formed Defects

As one can see in Figure 4b, three doubly occupied states are present in the bottom half of the band gap, similar to oxygen vacancies in crystal.^[^ ^58^ ^]^ In Figure 4c, the projected density of states (PDOS) and the inverse participation ratio (IPR) of the one‐electron spectrum are also plotted. Calculations of IPR exploit the atom‐centered basis set to quantify the degree of localization of a one‐electron state (see, refs. [121, 122, 123]). Higher values correspond to more localized states, whereas fully delocalized states have small IPR values. The localization of valence band states due to disorder is often observed in amorphous oxides^[^ ^5^, ^57^ ^]^ and silicate glasses (see, e.g., ref. [124]). For localized electronic states, the charge density extends over only a few atoms, usually including low‐coordinated atoms. These states serve as precursors for hole trapping in amorphous oxides.^[^ ^5^, ^57^ ^]^ In our calculations shown in Figure 4c, the top of the valence band is set at 0 eV. It can be seen that the states at the top of the valence band and the bottom of the conduction band exhibit a degree of localization also observed in the previous work.^[^ ^99^ ^]^ The three localized states in the band gap seen at 1.0–2.0 eV above the top of the valence band in Figure 4c are the three defect states seen in Figure 4b but calculated at 0 K.

The existence of these localized states allows one to easily define the “defect” states in a‐Al_2_O_3_ and, by analyzing the geometric structure of the ions surrounding the localized state, to describe its structural model. We find that in all cases, the Al ions adjacent to the defect are 3C or 4C. Another structural feature common to all of the defects is the existence of at least one very short Al–Al distance of about 2.5–2.6 Å, regardless of whether or not the Al ions are predominantly 3C or 4C. Figure 2d illustrates how the interaction between the two Al atoms drives the formation of this defect state as the temperature decreases. At ≈2750 K, the Al–Al separation is reduced from about 7.5 Å  to below 3.0 Å, which coincides with the formation of the bond‐like state between the two Al atoms (see Figure 2e). Above this temperature, thermal fluctuations can break this interaction, but below it, the two Al ions remain bound to each other, vibrating about this short separation for the rest of the simulation. Figure 2e shows the distribution of the electron density in two localized defect states, which involve two and three Al neighbors.

In summary, in these M&Q AIMD simulations, electrons and ions have a better chance of finding more favorable positions with respect to static P&R calculations at 0 K. Remarkably, in all three models, electrons have formed localized states during cooling, and the two structural properties responsible for the localization of the defect electronic states are the under‐coordinated Al ions and the Al–Al bond formation.

### 5.3 Annealing of Existing Vacancies

The structures of defects associated with oxygen deficiency of a‐Al_2_O_3_ described above are markedly different from the usual P&R O vacancy structures produced by removing O atoms from 3C or 4C O sites in quenched amorphous structures (see Figure 3d,e). A natural question arises: What will happen to such P&R vacancies upon high‐*T* annealing? Will they remain stable or will they re‐arrange into the Al–Al bond type structures described above? To investigate that, we used three pseudo‐amorphous 360 atom cells produced using the forcefield^[^ ^125^, ^126^ ^]^ in ref. [99] using a low cooling rate of 1.0 Kps^−1^ and relaxed at 0 K using the PBE0‐TC‐LRC functional. After removing an O atom, three of such structures were subjected to NVT annealing at 1000 K for 20 ps. The original vacancies produce deep KS levels in the band gap with two electrons localized inside the vacant site.^[^ ^58^ ^]^

The anneal does not lead to dissolution of vacancies or delocalization of defect KS states. Rather, we observe how the amorphous structures are further relaxed as a consequence of anneal. This relaxation stems from two sources. First, the high‐*T* anneal allows the system to explore a local phase space and find a lower energy configuration for the given vacancies. Secondly, the vacancies may also be able to diffuse to nearby sites, and thereby find a new position with lower total energy. The first effect can be further divided into local and global contributions. Local contributions are significant relaxations that occur within the nearest and next‐nearest neighbors of the vacancy. The global changes occur as the system explores the energy landscape at high‐*T* and are not necessarily correlated to the presence of the vacancy.

**Figure 5:**
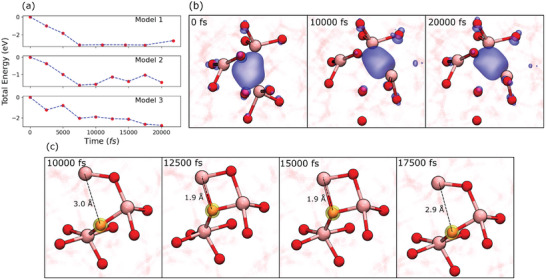
The energy and structure evolution of three periodic cells each containing one oxygen vacancy during a 1000 K anneal. Peach spheres show Al ions, red spheres show O ions. Blue surface indicates an iso‐surface of |ψ|^2^ of an in‐gap defect state. a) Each panel shows the total energy evolution of one of the 357‐atom models (Models 1‐3). The total energy is calculated by taking the configuration from AIMD at the time shown and then optimizing the atomic coordinates. b) Three geometry‐optimised snapshots of a post‐quench vacancy trajectory during the 1000 K anneal in AIMD. Initially, the vacancy is 3C, with |ψ|^2^ of the defect shared roughly equally between three adjacent Al ions. During the anneal, however, there is a significant displacement of an Al ion, producing a close Al–Al pair. This configuration remains for the rest of the anneal. c) Geometry‐optimized snapshots of ion motion during the 1000 K anneal. Initially, an O ion (highlighted) is 2C with respect to Al. After some time, it has moved to become 3C. This interim state eventually returns back into its original 2C position. The displayed Al–O distance illustrates the amount of the O ion displacement.

**Figure** **5**a shows the total energy evolution of the three models that undergo the 1000 K anneal. Each panel shows the energy of the system at several timesteps, after the geometry of the particular configuration corresponding to that time step has been additionally optimized. After a major relaxation lasting about 7.5 ps the structure approaches a new minimum and the energy stabilizes. The third panel in Figure 5a corresponds to a trajectory where we see not only relaxation of the structure, but also a vacancy migration event. One of the vacancies in the cell migrates by one atomic position, which is accompanied by a significant reduction of total energy. Thus, the defect migration in this system also contributes to structural relaxation, because vacancy sites are not equivalent in amorphous solids.

Another typical event can be seen in Figure 5b. Before the MD anneal begins, the vacancy is coordinated by three Al ions with the defect wavefunction iso‐surface distributed approximately evenly over these ions. After 10 ps, there is a significant Al ion displacement, leading to the reduction of the Al–Al separation (Figure 5b, middle panel). Correspondingly, the electron density has been redistributed and is now mainly shared between two Al ions. This configuration remains stable for the rest of the anneal. This 3C → 2C relaxation, accompanied by a redistribution of the electron density into a bond‐like state, is consistent with the above conclusion from in‐melt vacancies that the Al–Al bond formation by under‐coordinated Al ions creates the lower energy defects in amorphous alumina.

Further analysis of the anneal process shows several interesting events that cannot be observed when performing the usual geometry optimization. There are neighbor exchange events, where adjacent Al polyhedra exchange oxygen ions. These exchange events never result in the creation of a 3C Al ions, as the neighboring O ions and Al ions always displaced so as to maintain stoichiometry‐consistent CNs. An example of such behavior is seen in Figure 5a (Model 2), where the total energy exhibits oscillations after initial stabilization during 7.5 ps anneal. These oscillations correspond to significant displacements of an O ion seen in Figure 5c. An alternative way of understanding this structural rearrangement during annealing is an oxygen exchange with a 3C Al polyhedron. We also find the Al exchange between O ions during the annealing. Similar to O exchange, this never causes the formation of a coordination defect as the resulting structure does not contain O ions with a coordination number below 2. Some of these Al exchange events involve the 3C Al ions that belong to the defect.

The “reversibility” of defect states upon carrier trapping/de‐trapping is an important feature of stable defects in crystals, where, in most cases, the oxygen vacancy will regain the same structure and electronic level in the process of ionization and subsequent re‐trapping of an electron accompanied by the full defect relaxation. In amorphous oxides, some defect states have been predicted to have irreversible character because of global structural changes in the amorphous cell induced by the introduction of a defect or by the change of its charge state. For example, in ref. [70] it was found that ionizing the Me−Me bond to the +2 charge state leads to displacements of atoms in the unit cell, eliminating localized states associated with a point defect. These changes are found to be irreversible—upon re‐introducing two electrons back into the structure, according to ref. [70], the Me−Me bonds in a‐IGZO will not re‐form, and the electrons stay at the bottom of the conduction band, which is a more stable configuration. Similar behavior has been observed in a‐Al_2_O_3_ in ref. [68]. Whether oxygen vacancy can serve as an electron trap in a‐Al_2_O_3_ and other amorphous oxides is a fundamental issue for many applications. In our case, the Al–Al bond is also broken upon ionization into the +2 charge state, causing large displacements of the Al ions. The separation in the Al–Al pair increases by 0.5–1.2 Å, depending on the local environment. Once the geometry is converged, there is no localized state associated with the defect in the band gap. However, unlike in ref. [68], the re‐introduction of two electrons always causes an Al–Al bond restoration and the bond‐like electronic state to re‐form. Thus, in our calculations, these defect states are reversible and can participate in electron trapping/detrapping processes.

These results contradict the conclusions of refs. [68, 70, 82] that the oxygen vacancy is completely “assimilated” upon 2000 K anneal by the amorphous structure in a‐Al_2_O_3_, a‐HfO_2_ and a‐IGZO with no states in the band gap in the neutral state. This means that the absence of an oxygen atom in the cell cannot be structurally distinguished from a disorder of atomic positions, and electrons usually associated with an oxygen vacancy are delocalized in the conduction band. One of the possible reasons for the discrepancy with^[^ ^68^ ^]^ is that in those simulations, MD trajectories were calculated with a GGA XC functional and a hybrid functional was only used to recalculate the electron density at snapshots of the MD trajectory. Since the hybrid functional is not used during the trajectory calculation (e.g., to calculate forces), the self‐interaction is not canceled out, and hence the full coupling between electrons and nuclei is not accounted for ref. [127]. The vacancy electrons are delocalized at high temperatures due to the reduced band gap in GGA and do not localize in HSE calculations after cooling at 0 K. Apart from these differences, our periodic amorphous cell of 360 atoms is much larger than the 160 atom cell used in ref. [68] and we carried out calculations for three different cells and nine vacancies, unlike a single calculation in ref. [68]. These results illustrate the sensitivity of these predictions to the choice of XC functional and other computational parameters, mainly caused by the polaronic character of the electron localization accompanying this defect formation.

## 6 Discussion and Outlook

We discussed the meaning and origins of oxygen vacancy models in oxide crystals and whether these can be transferred to amorphous oxides. Understanding defects in amorphous materials is a timely problem, the complexity of which is under‐appreciated by the community. For example, many previous studies have used the P&R method to create atomistic “vacancy” models using the methodology borrowed from crystal studies and found similarities between the two cases. The experimental data on the structure and properties of defects are circumstantial and require complex analysis and modeling. Several theoretical studies that did not follow the traditional approaches arrived, however, with contradictory conclusions regarding the structure and stability of oxygen deficient states. The fundamental questions are how defects caused by oxygen deficiency or interstitial ions manifest themselves in amorphous systems and whether transferring models developed in crystals are sufficient to describe them. Our own results and those of the literature simulations show that these models are not sufficient and that other types of defect structures are possible, which are specific to amorphous structures. However, most of the atomistic defect models in amorphous oxides come from complex computer simulations, which involve many approximations, which are fundamental for DFT and other available techniques. We discussed some of the limitations of these computational methods, but we cannot offer a perfect solution to all of them.

To test and possibly overcome some of these limitations, we used AIMD with a hybrid XC functional and larger periodic cells to model the electronic properties of oxygen‐deficient amorphous alumina. In our melt and quench NPT‐ensemble simulations, no similarity to well‐defined crystallographic vacancies has been assumed. By melting and cooling substoichiometric Al_2_O_3_ models in AIMD, we have found stable point defect structures with deep, localized electronic states. These structures are different from those obtained using the P&R method and are characterized by the formation of Al–Al bonds between low‐coordinated Al ions sharing two electrons. To further investigate the validity of P&R models for oxygen deficiency in amorphous oxides, we subjected a number of P&R models to an anneal in AIMD. We find that Al–Al bonds are more stable defect configurations than relaxed vacancies produced by removing oxygen atoms at different positions in the amorphous structure. They form deep states with similar properties to oxygen vacancies in a crystal, and P&R vacancies in amorphous oxides. The annealing of pre‐existing vacancies thus confirms the stability of Al–Al bond configurations. We note the similarity of these structures to neutral vacancies in crystalline and amorphous SiO_2_^[^ ^73^ ^]^ and IGZO,^[^ ^70^ ^]^ where the metal–metal bond formation is associated with oxygen deficient defects. Thus, these results demonstrate that “point defect” is still a meaningful concept in disordered systems, but some configurations do not have the same meaning as a crystallographic defect. We note that the formation and strength of Al–Al and more generally T–T (T = B, Al, Ga, In) bonds is a well‐established concept in organic molecules (see e.g. refs. [128, 129]) and these bonds are the subject of active research. Interestingly, the Al–Al separations found in our simulations are very close to those in organic compounds,^[^ ^128^, ^129^ ^]^ confirming the analogy.

Our results, however, also suffer from many limitations, such as the melt cooling rate, cells size, density functional, statistical sampling, etc. and are by no means perfect. They serve to highlight the problems of defining and modeling defects in amorphous materials, such as oxide films. A question arises: are there other, more accurate, and efficient ways to investigate oxygen‐deficient amorphous oxide structures without imposing concepts that are only meaningful in crystal physics at reduced computer costs to improve statistics? Let us consider the main factors that affect the accuracy and costs of these simulations, such as the cooling rate of AIMD, XC functional, and statistical sampling.

Due to the computational expense of AIMD with a non‐local XC functional, we have resorted to using a high cooling rate of 100 Kp s^−1^ during the quench phase of the simulation. Compared to previous studies, this is several times higher than what is used in classical MD M&Q simulations. However, this is similar to the quench rates used to study amorphous oxides in other work,^[^ ^130^ ^]^ where the results did not differ significantly when the quench rate was decreased to 0.1 Kp s^−1^. Other studies^[^ ^131^ ^]^ that investigate AIMD M&Q simulations for producing HfO_2_ and ZrO_2_ have used even higher quench rates (500 Kp s^−1^) and shorter melt equilibration times and found that the coordination number distributions are distinct from those produced in classical MD, which utilizes much lower quench rates and much longer equilibration times. The literature data therefore suggest that the high quenching rate of 100 Kp s^−1^ is at the limit of what can produce reasonable structures, as judged by the coordination number distributions and density. As our results demonstrate, frozen melts produced at such high cooling rates are still far from annealed amorphous structures, and defect‐induced perturbations induce both local and global changes in their structure. As each defect site in the amorphous structure is unique, finding all local ground state configurations^[^ ^132^ ^]^ is computationally challenging, and more defect configurations can be expected in alumina and other oxides. These configurations depend on the global structure of the amorphous oxide and may correspond to different stages of the evolution of the amorphous structure when exposed to anneal, bias application, carrier transport, and thermal stress in devices. A particularly complex situation corresponds to strongly reduced oxide films usually designated MeO_*x*_ (for example, SiO_*x*_,^[^ ^133^ ^]^ TaO_*x*_, HfO_*x*_, TiO_*x*_^[^ ^134^ ^]^ and others), in which oxygen deficiency can lead to metallization and the mobile species is thought to be metal cations.

Describing reliably the electron localization in DFT is a much more complicated issue (see discussion in, e.g., refs. [101, 103]). The many‐body self‐interaction of polarons requires using piecewise‐linear functionals. Recently, there have been significant advances in applications of a more efficient DFT+U method to describe polaron localization, properties, and hopping in several oxides.^[^ ^103^, ^135^, ^136^, ^137^, ^138^ ^]^ Further developments of this approach may open opportunities for much faster AIMD screening for defect geometries.

Finally, we note that sampling of different sites in amorphous structures plays a very important role in determining both the probabilities of the formation of defect configurations and the distributions of their properties. Due to the large computational expense required for exhaustive sampling, issues related to statistical sampling are rarely discussed in the literature.^[^ ^53^, ^57^, ^60^ ^]^ However, poor sampling can significantly skew the predictions of these simulations. These calculations can be aided by the further development of new machine learning approaches to model electron localization and polaron states.^[^ ^139^ ^]^

In summary, understanding defects in amorphous structures is a growing area of research that continues to expose challenging problems in computational materials science. In this perspective, we have reviewed some of the concepts and challenges that exist in experimental studies and atomistic modeling of defects in amorphous oxides. The ill‐defined nature of defects in amorphous systems can lead to an improper description of the configuration of oxygen‐deficient structures in amorphous oxides. Some previous attempts to deal with these issues have been stifled by problems with the description of physical interaction (using semi‐local XC functionals, for example). Our results suggest that a point defect description of oxygen‐deficient structures is valid in the case of a‐Al_2_O_3_ and emphasize the importance of exploring alternative atomistic models of oxygen‐deficient structures, such as the formation of Me–Me dimers. These results demonstrate that our understanding of defects in amorphous oxides is still very far from complete, and we hope that this perspective will inspire further debate and research in this field.

## 7 Experimental Section

Here, the density functional theory (DFT) calculations used the CP2K Quickstep code,^[^ ^140^ ^]^ which employed a primary linear combination of atomic orbitals (LCAO) basis set with an auxilliary planewave basis set. The Goedecker–Teter–Hutter (GTH) pseudopotentials and basis sets were used.^[^ ^141^, ^142^ ^]^ A double‐ζ basis set was used for the valence electrons in all cases, with higher *l* orbitals used for polarization basis functions, and an auxiliary planewave basis set has a cutoff of 7875 eV. The hybrid functional PBE0‐TC‐LRC^[^ ^143^ ^]^ was used to calculate the exchange‐correlation (XC) energy. The parameters of this functional were tuned to enforce the piece‐wise linearity with respect to fractional particle occupations, as described in ref. [99]. The auxiliary density matrix method (ADMM)^[^ ^144^ ^]^ improved the efficiency of calculations. All calculations were performed at the Γ point. In the calculation of defect charge transition levels, the charge correction method from ref. [145], which was based on the Lany–Zunger method was used.^[^ ^146^ ^]^ Due to computer time limitations, all AIMD calculations were performed in the singlet spin state.

The first set of AIMD simulations (melt and quench) used the NPT ensemble, with the pressure set to 1 atm and a timestep of 0.5 fs. To reduce the computational cost and enhance the equilibration time, the cell was first equilibrated at 4000 K with the PBE functional for 12 ps, followed by a 3 ps PBE0‐TC‐LRC equilibration run. After the equilibration, the system was quenched at the rate of 100 Kp s^−1^ down to 0 K using PBE0‐TC‐LRC. In the second set of MD simulations (annealing of vacancies), the a‐Al_2_O_3_ models generated in ref. [58] were annealed with single O vacancies introduced by the P&R method for 20 ps in the NVT ensemble at a temperature of 1000 K using the PBE0‐TC‐LRC functional.

For both NPT and NVT simulations, a CSVR^[^ ^147^ ^]^ thermostat was used, and the NPT simulations employed a Nose–Hoover barostat with a target pressure of 1 atm. Using the NPT ensemble better reflected the formation of amorphous oxides from high‐*T* melt. In NVT calculations, the cell parameters were optimized first in static calculations. Once oxygen atoms were deleted, the cell parameters were kept fixed, and only the atomic positions within the cell were varied to minimize potential energy.

## Conflict of Interest

The authors declare no conflict of interest.
